# Carnitine metabolites and cognitive improvement in patients with schizophrenia treated with olanzapine: a prospective longitudinal study

**DOI:** 10.3389/fphar.2023.1255501

**Published:** 2023-08-17

**Authors:** Lei Zhao, Hua Liu, Wenjuan Wang, Youping Wang, Meihong Xiu, Shuyun Li

**Affiliations:** ^1^ Qingdao Mental Health Center, Qingdao, Shandong, China; ^2^ Department of Nutritional and Metabolic Psychiatry, The Affiliated Brain Hospital of Guangzhou Medical University, Guangzhou, China; ^3^ Guangdong Engineering Technology Research Center for Translational Medicine of Mental Disorders, Guangzhou, China; ^4^ Key Laboratory of Neurogenetics and Channelopathies of Guangdong Province and the Ministry of Education of China, Guangzhou Medical University, Guangzhou, China; ^5^ Beijing HuiLongGuan Hospital, Peking University HuiLongGuan Clinical Medical School, Beijing, China

**Keywords:** schizophrenia, carnitines, cognitive improvement, olanzapine, longitudinal study

## Abstract

**Objective:** Cognitive impairment is one of the core symptoms of schizophrenia, which is stable and lifelong. L-carnitine has been shown to improve cognitive function and decrease the rate of cognitive deterioration in patients with Alzheimer’s disease. However, it remains unclear regarding the role of L-carnitine and its metabolites in cognitive functions in schizophrenia after treatment with olanzapine. The purpose of this study was to evaluate the relationship between changes in plasma levels of L-carnitine metabolites and cognitive improvement after olanzapine treatment.

**Methods:** This was a prospective longitudinal study. In this study, we recruited 25 female patients with first episode schizophrenia (FES) who were drug naïve at baseline and received 4 weeks of olanzapine monotherapy. Cognitive function was assessed at baseline and 4-week follow-up using the RBANS. Plasma L-carnitine metabolite levels were determined by a metabolomics technology based on untargeted ultra-performance liquid chromatography-mass spectrometry (UPLC-MS).

**Results:** We found that the immediate memory index, delayed memory index and RBANS composite score were significantly increased at the 4-week follow-up after treatment. A total of 7 differential L-carnitine metabolites were identified in FES patients after olanzapine monotherapy. In addition, we found that changes in butyrylcarnitine were positively correlated with improvements in language index and RBANS composite score. Further regression analyses confirmed the association between reduced butyrylcarnitine levels and cognitive improvement after olanzapine monotherapy in FES patients.

**Conclusion:** Our study shows that cognitive improvement after olanzapine treatment was associated with changes in L-carnitine metabolite levels in patients with FES, suggesting a key role of L-carnitine in cognition in schizophrenia.

## 1 Introduction

Patients with schizophrenia are characterized by modest to severe impairments in verbal learning, working memory, executive function and processing speed (Barnett, 2018; Harvey and Isner, 2020; Xiu et al., 2020; Xiu et al., 2021; Zhu et al., 2022). Cognitive impairment is present throughout the course of the disorder, from prodromal to more severe stages. Large retrospective cohort studies showed that cognitive impairment is the first sign and a trait marker in individuals later diagnosed with schizophrenia (Häfner et al., 1992; Rund, 1998). In addition, evidence of cognitive impairment in the prodromal stage and throughout the course of schizophrenia demonstrates a close relationship between cognitive impairment and independent living, social and community cognitive function, and functional outcomes (Green and Nuechterlein, 1999; Bilder et al., 2000; Green et al., 2000; Fett et al., 2011).

Carnitine, biosynthesized from an amino acid, is present in nearly all cells of the body (Flanagan et al., 2010). There are two forms of carnitine, known as D-carnitine and L-carnitine, and only L-carnitine is active in the body. L-carnitine is found in mammalian cells as free carnitine and acylcarnitines. *In vivo*, carnitine can be transferred with the acyl group by carnitine palmitoyltransferase 1 to produce acylcarnitine (Bonnefont et al., 2004). L-carnitine has various biological functions, such as in the metabolism of fatty acids as energy to keep the body’s cells powered and working efficiently and in anti-inflammatory and antioxidant defense (Moghaddas and Dashti-Khavidaki, 2016; Traina, 2016). For example, the L-carnitine system is known for its role in the transport of fatty acids into the mitochondrial matrix and β-oxidation of fatty acids (Noland et al., 2009). Cellular energy production is primarily derived from mitochondrial β-oxidation of fatty acids, especially when carbohydrate stores are exhausted after exercise. L-carnitine can improve mitochondrial and peroxisomal metabolism in neurons (Jones et al., 2010). Abnormal levels of L-carnitine or its metabolites may decrease fatty acid β-oxidation and reduce the production of mitochondrial energy in mental disorders (Kępka et al., 2021). In addition, L-carnitine maintains the integrity of cell membranes and stabilizes the physiological CoA-SH/acetyl-CoA ratio in mitochondria (Siliprandi et al., 1990). Its deficiency causes the structural swells of astrocytes and the expansion of mitochondria in nerve cells. Noteworthy, impaired mitochondrial function in schizophrenia is supported by converging evidence from genetic, post-mortem and peripheral studies (Clay et al., 2011; Rajasekaran et al., 2015). Interestingly, compounds containing L-carnitine substructures did show neuroprotective effects, which were reported to be related to the protection of mitochondria accompanied by improved energy supply (Spagnoli et al., 1991; Wang et al., 2017).

Antipsychotics are the first-line treatment for patients with schizophrenia and there is evidence that olanzapine (OLA) improves cognitive function in patients with schizophrenia (Ljubin et al., 2000; Sergi et al., 2007; Baldez et al., 2021), although the effect size was small and some prospective cohort studies have reported inconsistent results (Baldez et al., 2021). For example, the Clinical Antipsychotic Trials of Intervention Effectiveness (CATIE), a clinical trial with one of the largest neuropsychological tests in schizophrenia, showed moderate improvements in patients following antipsychotic drugs (Keefe et al., 2007). On the contrary, a naturalistic sub-group analysis demonstrated that discontinuation of antipsychotic medication was not associated with a negative effect on cognitive function, but with a better effect on it (Albert et al., 2019). Cognitive impairments in schizophrenia have been shown to be related to abnormalities in several biological pathways (Xiu et al., 2019; Wu et al., 2020; Xiu et al., 2020; Su et al., 2021). Of particular interest is the accumulating evidence of carnitine deficiency in cognitive deficits in general populations, which may also be a possible pathological mechanism of cognitive impairments in schizophrenia. Given the fundamental role of L-carnitine in mitochondrial functions and energy production, a growing body of evidence supports its dysfunctions in cognitive declines, such as Alzheimer’s disease (AD) (Signorelli et al., 2006; Ciacci et al., 2007; Wesnes and Reynolds, 2019; Pennisi et al., 2020; Kępka et al., 2021).

Moreover, abnormal L-carnitine levels may be associated with certain mental illnesses, including schizophrenia and depression (Wang et al., 2014; Kriisa et al., 2017). Current evidence supports an imbalance in the oxidative stress status or inflammatory status involved in the pathogenesis of schizophrenia, which is also related to β-oxidation in the mitochondrion of L-carnitines (Cuturic et al., 2016). In addition, acetyl-carnitine supplementation to clozapine therapy has been shown to improve positive symptoms in patients with schizophrenia. Together, all these studies suggest that carnitine or its metabolites play a pivotal role in the treatment response of schizophrenia.

We hypothesized that abnormal levels of L-carnitine metabolites are associated with cognitive impairments and that changes in them after OLA monotherapy are associated with cognitive improvement in FES patients. Therefore, to test this hypothesis, we recruited unmedicated FES patients and conducted a comprehensive analysis of L-carnitine metabolites in a 4-week OLA-treatment population with schizophrenia. We tested the following questions in schizophrenia: a) differences in L-carnitine metabolite levels in patients after OLA monotherapy relative to baseline; b) the relationship between changes in L-carnitine metabolites levels and cognitive improvement; and c) the predictive role of baseline L-carnitine metabolites levels for cognitive improvement.

## 2 Methods

### 2.1 Patients

This study was conducted from July 2011 to January 2013. The study protocol was reviewed and approved by the Institutional Review Board of Beijing Huilongguan Hospital. Patients signed the written informed consent forms.

A total of 25 FES patients were recruited at Beijing Huilongguan Hospital. The definition of FES was as a previous study (Lieberman et al., 2003). The inclusion criteria included: 1) diagnosis of schizophrenia by the Chinese version of the Structured Clinical Interview (SCID) for Diagnostic and Statistical Manual of Mental Disorders IV (Phillips and Liu, 2011); 2) medication naïve; 3) age between 18 and 45 years old; and 4) experiencing a first episode of psychosis. The exclusion criteria included: 1) abuse or substance dependence obtained through the questions we asked participants verbally; 2) suicide ideation; 3) pregnancy or breastfeeding; 4) serious neurological or major medical illnesses; and 5) receiving antidiabetic, antihyperlipidemic, and/or antihypertensive drugs.

### 2.2 Study procedures

A prospective, observational, cohort study with a 4-week follow-up was conducted on patients with schizophrenia. A questionnaire was designed to collect demographic and clinical data. Over the course of 4weeks of treatment, FES patients received a flexible-dosage, oral OLA monotherapy as prescribed by the psychiatrists based on clinical response. The oral dose of OLA for FES patients ranges from 10 mg/day to 30 mg/day. During the 4 weeks, all patients were hospitalized and nurses monitored OLA medication adherence.

### 2.3 Assessment

The Repeatable Battery for the Assessment of Neuropsychological Status (RBANS, Form A) was used to assess the cognitive functioning of patients (Randolph et al., 1998). The index score of the RBANS includes immediate memory, visuoconstructional, attention, language, and delayed memory. All these index scores are combined to form a composite score. In addition, the Positive and Negative Syndrome Scales (PANSS) were evaluated to determine the severity of clinical symptoms (Kay et al., 1987). After a brief standardized training, repeated assessments showed that the inter-rater reliability of the PANSS total score maintained greater than 0.8. Cognitive functions and clinical symptoms were assessed at baseline and at the end of 4 weeks.

### 2.4 Plasma collection and metabolomics processing

Fasting blood was collected by the research nurse at 7:00 a.m. at baseline and at the 4-week follow-up. Plasma samples were separated and 200 μl was ground into powder in liquid nitrogen. As reported in our previous study, an LC-HRMS system, Q-Exactive Focus equipped with a heated electrospray ionization source was used for untargeted metabolomics (Liu J et al., 2021; Liu J H et al., 2021).

### 2.5 Statistical analysis

The concentrations of L-carnitine metabolites were not normally distributed, thus, we performed the non-parametric tests in this study. Non-parametric analyses of paired sample t-test were used to compare clinical symptoms and cognitive functions at baseline and 4-week follow-up. Spearman rank correlation analysis was used to evaluate the association between L-carnitine metabolites and cognitive improvements in patients. In addition, we divided the original α-values by the number of analyses performed on the variables to obtain Bonferroni-corrected/adjusted p values. The new α = 0.05/7 = 0.007 for metabolite analysis and α = 0.05/6 = 0.008 for cognitive function analysis. Multiple linear regression analysis was performed to investigate the influencing factors for cognitive improvement in FES patients, by limiting the effects of confounding factors, such as age, education and baseline BMI. Improvements in RBANS total score or its subscores served as dependent variables and changes in L-carnitine levels or baseline L-carnitine levels served as independent variables in the current study.

Data were analyzed using statistical software (IBM SPSS 22.0). The significance threshold was set at *p* < 0.05.

## 3 Results

The demographic and clinical characteristics of the FES patients are shown in Sup Table 1. As reported in our previous study, OLA treatment for 4 weeks significantly improved clinical symptoms and cognitive functions in patients with FES (p_Bonferroni_ < 0.05).

**TABLE 1:** Demographic and clinical variables of patients at baseline and at follow-up.

Variables, mean ± SD	First-episode	After 4 weeks	
	n = 25	n = 25	t(p)
Age (ys)	27.4 ± 7.6		
Education (ys)	9.1 ± 3.5		
BMI (kg/m2)	21.0 ± 3.6		
Age of onset	26.4 ± 8.9		
PANSS total score	81.8 ± 13.9	63.9 ± 13.8	5.4 (<0.001)
P subscore	25.0 ± 6.0	16.4 ± 5.2	7.6 (<0.001)
N subscore	17.1 ± 4.7	15.6 ± 4.3	1.2 (0.23)
G subscore	39.7 ± 7.5	32.0 ± 6.0	4.2 (<0.001)

**Abbreviations:** ys years; BMI, body mass index; PANSS, positive and negative syndrome scale; P subscore, positive symptom subscore; N subscore, negative symptom subscore; G subscore, general psychopathology subscore.

### 3.1 L-carnitine metabolites determination

Plasma metabolomics analysis identified abnormalities in lipids, organic acids, bilirubin, carnitine, ammonium salts, proline, olanzapine and its metabolites after treatment. Among these compounds, we identified 7 differential L-carnitine metabolites with a VIP value >1 and *p* < 0.05. Further analysis showed that 2-Octenoylcarnitine, 11Z-Octadecenylcarnitine, 9-Decenoylcarnitine and L-Palmitoylcarnitine levels were significantly decreased after OLA monotherapy relative to baseline levels (all *p* < 0.05) (Table 2). In contrast, there were no significant differences in the levels of other 3 L-carnitine metabolites between baseline and follow-up (all *p* > 0.05). There was no significant association between plasma levels of L-carnitine metabolites and RBANS total score or its five subscores at baseline.

**TABLE 2:** Carnitines of the FES patients at baseline and after 4 weeks.

Carnitine, median (IQR)	First-episode (10^4^)	After 4 weeks (10^4^)	Z(p)
Linoelaidyl carnitine	9.2 (3.1, 327.4)	54.0 (39.2, 71.8)	−0.3 (0.82)
2-Octenoylcarnitine	164.9 (83.5, 321.5)	4.9 (2.7, 10.9)	−3.8 (<0.001)
Butyrylcarnitine	94.9 (58.7, 230.5)	125.3 (69.6, 224.5)	−1.3 (0.20)
11Z-Octadecenylcarnitine	14.9 (2.3, 189.1)	203.4 (129.2, 317.3)	−2.2 (0.03)
9-Decenoylcarnitine	3.2 (2.2, 4.5)	14.5 (1.8, 194.8)	−2.9 (0.004)
Propionylcarnitine	38.5 (14.0, 72.8)	30.0 (13.2, 55.1)	−1.4 (0.17)
L-Palmitoylcarnitine	89.0 (35.4, 279.3)	3.5 (2.8, 4.0)	−4.4 (<0.001)

### 3.2 Association of baseline L-carnitine metabolites with cognitive improvement

Spearman correlation analysis showed that baseline 9-Decenoylcarnitine levels were significantly associated with improvements in the delayed memory index (r = 0.43, *p* = 0.031) (Table 3). However, this association did not pass the Bonferroni correction. After controlling for onset age, education and baseline BMI, multiple regression analysis also did not find a significant association between baseline metabolite levels and cognitive improvement with cognitive improvement as the independent variable and baseline 9-Decenoylcarnitine level as the dependent variable (*p* > 0.05).

**TABLE 3:** Correlations of baseline lysophosphatidylcholine levels and the improvement in cognitive functions in patients.

Baseline carnitines	Immediate memory (r/p)	Visuoconstructional (r/p)	Language (r/p)	Attention (r/p)	Delayed memory (r/p)	Total score (r/p)
Linoelaidyl carnitine	−0.16 (0.44)	0.13 (0.54)	0.04 (0.84)	−0.19 (0.37)	−0.12 (0.55)	−0.21 (0.31)
2-Octenoylcarnitine	0.36 (0.08)	0.39 (0.05)	−0.06 (0.78)	−0.06 (0.77)	0.11 (0.61)	−0.05 (0.80)
Butyrylcarnitine	0.14 (0.50)	0.22 (0.29)	0.30 (0.15)	−0.04 (0.85)	0.31 (0.14)	0.20 (0.34)
11Z-Octadecenylcarnitine	−0.15 (0.48)	0.17 (0.41)	0.24 (0.26)	−0.28 (0.18)	0.02 (0.94)	0.06 (0.78)
9-Decenoylcarnitine	0.15 (0.48)	0.11 (0.61)	0.20 (0.33)	0.14 (0.49)	**0.43(0.03)**	0.30 (0.15)
Propionylcarnitine	0.27 (0.19)	0.36 (0.08)	0.17 (0.43)	0.02 (0.93)	0.14 (0.50)	0.10 (0.64)
L-Palmitoylcarnitine	0.05 (0.81)	0.23 (0.26)	0.03 (0.89)	−0.22 (0.28)	−0.004 (0.9)	−0.10 (0.62)

Note: The bold *p* value means less than 0.05.

### 3.3 Association between changes in plasma L-carnitine metabolite levels and cognitive improvement

As shown in Table 4, we found that the decrease in 2-octenoylcarnitine levels was associated with the improvement in immediate memory index (r = −0.43, *p* = 0.033). In addition, the decrease in butyrylcarnitine was positively correlated with improvements in language performance (r = 0.47, *p* = 0.017) and RBANS composite score (r = 0.42, *p* = 0.039). Further regression analysis also showed a significant association between a decrease in butyrylcarnitine and improvement in language index (β= 0.49, t = 2.5, *p* = 0.021) or RBANS composite score (β= 0.45, t = 2.3, *p* = 0.034) after controlling for onset age, education and baseline BMI.

**TABLE 4:** Correlations of the changes in carnitine levels and cognitive improvement in FES patients.

Decreases in carnitines	Immediate memory (r/p)	Visuoconstructional (r/p)	Language (r/p)	Attention (r/p)	Delayed memory (r/p)	Total score (r/p)
Linoelaidyl carnitine	−0.16 (0.45)	0.15 (0.49)	0.04 (0.85)	−0.19 (0.35)	−0.12 (0.55)	−0.21 (0.32)
2-Octenoylcarnitine	**0.43(0.03)**	0.28 (0.17)	−0.11 (0.61)	−0.04 (0.84)	0.22 (0.30)	−0.01 (0.97)
Butyrylcarnitine	0.10 (0.64)	0.07 (0.74)	**0.47(0.017)**	0.26 (0.22)	0.36 (0.08)	**0.42(0.04)**
11Z-Octadecenylcarnitine	−0.23 (0.27)	0.06 (0.76)	0.11 (0.60)	−0.35 (0.09)	−0.27 (0.20)	−0.09 (0.66)
9-Decenoylcarnitine	0.19 (0.37)	0.08 (0.71)	−0.03 (0.90)	0.25 (0.22)	0.22 (0.29)	0.19 (0.36)
Propionylcarnitine	−0.12 (0.58)	0.23 (0.28)	0.26 (0.22)	−0.02 (0.92)	−0.35 (0.09)	−0.02 (0.95)
L-Palmitoylcarnitine	0.05 (0.81)	0.24 (0.26)	0.03 (0.88)	0.22 (0.29)	−0.003 (0.9)	−0.10 (0.62)

Note: The bold *p* value means less than 0.05.

## 4 Discussion

In this cohort study, we found that 1) OLA monotherapy significantly decreased plasma levels of four L-carnitine metabolites in FES patients; 2) reduced plasma levels of 2-octenoylcarnitine and butyrylcarnitine were correlated with the cognitive improvement after treatment; and 3) baseline L-carnitine metabolite levels were not associated with cognitive improvement.

We found that OLA significantly decreased several L-carnitine metabolites levels in FES patients, including 2-octenoylcarnitine, 11z-octadecenylcarnitine, 9-decenoylcarnitine and L-palmitoylcarnitine. All of these metabolites are known to be medium- or long-chain acylcarnitines with six or more carbons. More specifically, they are all acyl fatty acid derivative esters. In the body, acylcarnitines can be divided into nine categories based on the size and type of acyl group: 1) short-chain ACs; 2) medium-chain ACs; 3) long-chain ACs; 4) very long-chain ACs; 5) hydroxy ACs; 6) branched chain ACs; 7) unsaturated ACs; 8) dicarboxylic ACs and 9) miscellaneous ACs. Medium- and long-chain acylcarnitines are slightly less abundant in the body than short-chain acylcarnitines and are involved in the mitochondrial β-oxidation pathway in mitochondria. More specifically, 2-Octenoylcarnitine is an acylcarnitine having (2E)-octenoyl as the acyl substituent. 9-Decenoylcarnitine is an acyl fatty acid derivative ester formed by carnitine and arachidonic acid. L-palmitoylcarnitine is formed via palmitoyl-CoA reacts with L-carnitine, which is then moved into the mitochondrial intermembrane space. L-palmitoylcarnitine can react with the carnitine o-palmitoyltransferase 2 enzyme present in the mitochondrial inner membrane to once again form palmitoyl-CoA and L-carnitine. Palmitoyl-CoA then enters into the β-oxidation pathway to form acyl coenzyme A (aceytl-CoA). We found that levels of these three L-carnitine metabolites were decreased after treatment, which was in line with a previous study (Cao et al., 2019a), suggesting that OLA can downregulate acylcarnitine levels in patients with FES. However, given that no controls were recruited in this study, we do not know whether the decreased acylcarnitine levels normalized to those found in healthy controls, Acylcarnitine plays a fundamental role in the transfer of fatty acid to mitochondria for subsequent β-oxidation. Consistent with our findings, some studies have also reported abnormal levels of acylcarnitine in various diseases. For example, 9-decenoylcarnitine has been shown to be increased the plasma of overweight individuals (Kang et al., 2018). Metabolite profiling of the 1946 British birth cohort also found a relationship between a module consisting of acylcarnitine and processing speed after controlling for life course, showing L-palmitoylcarnitine to be a hub (Green et al., 2022). Prior studies have also found acylcarnitines levels with abnormalities in the plasma of patients with schizophrenia and familial Mediterranean fever (Kiykim et al., 2016; Cao et al., 2020). Moreover, the regulation of L-carnitine metabolites by antipsychotic drugs has been reported in clinical studies (Lheureux and Hantson, 2009; Molina et al., 2021; Yi et al., 2021), and in animal studies (Albaugh et al., 2012; Jiang et al., 2019), particularly in two early studies in patients with schizophrenia after treatment (Kriisa et al., 2017; Cao et al., 2019b). All these findings support our finding that the carnitine pathway can be regulated by OLA.

The second finding was that cognitive improvement after OLA treatment was significantly associated with decreased levels of 2-octenoylcarnitine and butyrylcarnitine in schizophrenia. And the lower the levels of 2-octenoylcarnitine and butyrylcarnitine, the better the cognitive improvement. This finding is the first report in patients with schizophrenia, but is consistent with recent findings in AD patients. Studies in AD and preclinical AD have shown that some medium- or long-chain acylcarnitines involved in fatty acid transportation and metabolism were associated with cognitive decline (Fiandaca et al., 2015; Ciavardelli et al., 2016). For example, plasma acylcarnitines have been found to decrease aging and have been shown to predict conversion to mild cognitive impairment or AD. In addition, altered metabolism of medium-chain acylcarnitines and impaired ketogenesis may be metabolic features of AD (Ciavardelli et al., 2016). We cannot give an exact explanation for the close relationship between the two special acylcarnitines and cognitive improvement. However, it is known that the carnitine shuttle pathway is responsible for the transport of long-chain fatty acids from the cytoplasm into the mitochondria for subsequent β-oxidation, a process that requires aceytl-CoA and leads to the esterification of L-carnitine to form acylcarnitine derivatives (Sharma and Black, 2009). It is possible that perturbation of the carnitine shuttle leads to compromised mitochondrial function, which could decrease cellular capacity to handle reactive oxygen species and increase the levels of the inflammatory cytokine, resulting in increased cellular dysfunction and cell death (Mitchell et al., 2018).

The brain is highly dependent on oxidative metabolism. In the absence of carnitine, fatty acid metabolism and energy production in the brain are impaired, leading to cognitive impairment. A previous review has supported the critical role of acylcarnitine in fatty acid metabolism, ketosis and buffering the concentration ratio of acyl-CoA to free CoA in brain metabolism in neurological disorders (Jones et al., 2010). Considering the close relationship between metabolic disturbances and acylcarnitine, we further analyzed the relationship between acylcarnitine and cognitive impairment after controlling for weight gain and found that the association between decreased acylcarnitine levels and cognitive improvement remained significant. Therefore, the association of cognitive improvement with acylcarnitine is reliable and robust, suggesting its role in schizophrenia.

There were several limitations to note in this study. First, only female patients were recruited in our study, as the majority of patients in our research center are female. Only female patients may limit generalizations to the broader population with FES schizophrenia. Second, we did not collect data on the levels of L-carnitine metabolites in the cerebro-spinal fluid (CSF). Further studies should include this data, which would lend consistency to the findings in this study.

In summary, we found that OLA treatment significantly decreased L-carnitine metabolite levels and improved cognitive functions in patients with schizophrenia. In addition, decreased carnitine metabolite levels were significantly associated with cognitive improvements after treatment. Our study provides new evidence for the involvement of L-carnitine metabolite levels in cognitive improvement after the treatment with OLA. However, due to the small sample size and the short-term OLA monotherapy, our findings should be interpreted with great caution. Moreover, only female patients may limit generalizations to the broader population with DNFE schizophrenia. Further longitudinal studies using larger samples with longer antipsychotic treatment are warranted to understand the exact mechanism of L-carnitine metabolites in cognitive improvement in schizophrenia.

## Data Availability

The raw data supporting the conclusion of this article will be made available by the authors, without undue reservation.
